# Multi-task learning for the simultaneous reconstruction of the human and mouse gene regulatory networks

**DOI:** 10.1038/s41598-020-78033-7

**Published:** 2020-12-18

**Authors:** Paolo Mignone, Gianvito Pio, Sašo Džeroski, Michelangelo Ceci

**Affiliations:** 1 Department of Computer Science, University of Bari Aldo Moro, Bari, 70125 Italy; 2 Department of Knowledge Technologies, Jožef Stefan Institute, Ljubljana, 1000 Slovenia

**Keywords:** Gene expression, Data mining, Gene regulatory networks, Machine learning

## Abstract

The reconstruction of Gene Regulatory Networks (GRNs) from gene expression data, supported by machine learning approaches, has received increasing attention in recent years. The task at hand is to identify regulatory links between genes in a network. However, existing methods often suffer when the number of labeled examples is low or when no negative examples are available. In this paper we propose a multi-task method that is able to simultaneously reconstruct the human and the mouse GRNs using the similarities between the two. This is done by exploiting, in a transfer learning approach, possible dependencies that may exist among them. Simultaneously, we solve the issues arising from the limited availability of examples of links by relying on a novel clustering-based approach, able to estimate the degree of certainty of unlabeled examples of links, so that they can be exploited during the training together with the labeled examples. Our experiments show that the proposed method can reconstruct both the human and the mouse GRNs more effectively compared to reconstructing each network separately. Moreover, it significantly outperforms three state-of-the-art transfer learning approaches that, analogously to our method, can exploit the knowledge coming from both organisms. Finally, a specific robustness analysis reveals that, even when the number of labeled examples is very low with respect to the number of unlabeled examples, the proposed method is almost always able to outperform its single-task counterpart.

## Introduction

Gene regulation is the process that allows a cell to express a particular group of genes and to inhibit others in specific contexts. For example, a nerve cell has the same genome as a muscle cell, but they are different because of the different sets of expressed genes in each of them. This explains how the cells of different tissues have different proteomes, that is, different sets of proteins produced as a result of the selective expression of a gene or a group of genes. Since tumor cells are mainly caused by the expression of genes outside the original context of the cell, the understanding of gene regulation mechanisms appears to be fundamental to study various forms of cancer^1,2^. In this context, the analysis of Gene Regulatory Networks (GRNs) appears to be a fundamental task.

A GRN represents the system of regulatory genes and their interactions that determine the genetic functions to be expressed in cells of each spatial domain in the organism, at every stage of development. This includes the expression of regulatory genes (i.e., genes encoding transcription factors), genes that encode intercellular signaling functions, and genes that participate in downstream differentiation and morphogenesis functions^3^. As stated by Smith et al.^4^, identifying the structure of GRNs helps in the biological understanding of disease mechanisms and increases possibilities for better medical/clinical care by improving diagnostics, prognostics and treatment. In particular, the reconstruction of a GRN comprises the identification of pairwise interactions between genes (i.e., nodes in the network) that participate in the same biological processes or that perform together specific biological functions that shape a system’s behavior and function^5^.

There are several techniques to elucidate the structure of a gene regulatory network. Some examples include ChIP-chip or ChIP-sequencing^6^, bacterial one-hybrid systems^7^ or protein-binding microarrays^8^. However, the validation process is often technically demanding, expensive and time-consuming^9^.

Alternatively, the task can be supported by computational approaches that analyze the expression levels of genes, measured under different conditions. Since the high availability of such data makes computational approaches affordable, there has been a significant increase in computational methods proposed in the literature^10–13^. The task at hand is also referred to as “reverse-engineering” or “gene network reconstruction”.

Existing methods usually do not rely on a single theory, but on multiple classes of statistical/mathematical methods and information/machine learning theory. In this context, the Dialogue for Reverse Engineering Assessments and Methods (DREAM) challenges have also contributed to the development of this task. In particular, in follow-up studies, it has been shown that combining multiple approaches^14,15^ or multiple sources^16,17^ can be beneficial for GRN reconstruction.

Considering Gene Network Reconstruction as a machine learning task, it can be formulated as a link prediction problem via binary classification, where existing relationships among genes (i.e., gene regulation activities that have already been validated in the laboratory) can be considered as the set of *positive examples*. On the other hand, pairs of genes, for which there is a confirmation about the non-existence of the regulation, can be considered as *negative examples*. However, validation efforts and resources are usually spent to prove the existence of gene interactions, rather than their non-existence. This means that all the possible gene pairs for which there is no web-lab validation cannot be considered negative examples, but rather *unlabeled examples*. This context makes the adoption of classical supervised machine learning methods inappropriate or even inapplicable, and requires the design of semi-supervised learning methods, which are also explicitly able to work in the absence of negative examples, i.e., in the *positive-unlabeled* setting^17^. This is the most challenging setting, especially considering that the number of available positive examples is usually significantly lower than the number of unlabeled examples.

In order to face the above challenges, in this paper we propose a machine learning method for gene network reconstruction, which works in the positive-unlabeled setting and alleviates the issues arising from the limited availability of labeled data. In particular, the method proposed in this paper relies on a *transfer learning* approach that is able to exploit the knowledge of a source domain $D_s$ to improve the result of a task performed on the target domain $D_t$. In our case, the data in each domain represents the expression levels measured for genes of a different organism.

Methodologically, we propose a specific kind of transfer learning approach, namely, a *multi-task* method^18^, whose main advantage is the ability to simultaneously solve the task on both domains, and possibly exploit dependencies between them that could lead to improved accuracy of reconstruction. In particular, we aim at simultaneously reconstructing the gene regulatory networks of two related organisms, namely, the human and the mouse regulatory networks, by considering a novel instance mapping which is guided by the notion of genetic orthology^19^.

State-of-the-art supervised machine learning methods employ a training set of examples which represents a sample of the population under analysis, described by a feature vector and associated with a known target value. These methods learn a prediction model which is able to assign a target value to unseen examples. This approach is widely proven to be effective if the set of examples is large enough and if the dataset is completely labeled, i.e., each example has a label (target value).

Therefore, classical supervised machine learning algorithms can be naturally applied to solve the task of network reconstruction, where: (i) each example corresponds to a (possible) relationship between two genes; (ii) features correspond to expression data regarding the two genes; (iii) labels can be {*Yes*, *No*}, depending on whether the interaction exists or not, or a numerical value representing the degree of certainty of the interaction. However, the quality of the reconstruction can be affected by the poor availability of labeled examples. Moreover, in this specific application domain, the available examples are usually only positive, i.e., they are only examples of existing interactions (see, for example, the well-known database BioGRID^20^, that contains only existing gene regulations, without any examples of verified non-existing ones).

In the literature, we can find different approaches to face this challenge, that usually work in the positive-unlabeled learning setting. They can be classified according to three categories^21^:

- *two-step methods*, that identify a set of negative examples from the set of unlabeled examples and then, in the second step, exploit off-the-shelf supervised learning methods to build the final predictive model^22–24^;
- *instance-weighting methods*, that estimate the reliability of each unlabeled example and exploit it as a weight or a cost while learning the prediction model^25^;
- *noisy negative methods*, that consider the unlabeled set of examples as highly noisy negative examples^22,26^.

The method proposed in this paper partially falls in category (b), that, according to previous studies^15,27^, allows us to avoid the imposition of strong assumptions on the negative examples, made by the methods in categories (a) and (c). However, as we explain in detail in “Methods” section, the estimated weight is used as a target value, rather than as a weight. Moreover, as introduced in “Introduction” section, the proposed method is based on a multi-task approach which simultaneously solves the network reconstruction task for two organisms, namely, human and mouse, possibly exploiting dependencies and similarities among them.

Since the method proposed in this paper solves the gene network reconstruction task by exploiting a *transfer learning* approach, specifically based on *multi-task learning*, in the following subsections, we provide some background notions and briefly review existing methods in these fields.

### Transfer learning

One possible solution to overcome the scarcity of labeled examples is the adoption of transfer learning approaches^18,28^, that aim at exploiting the knowledge about another related domain $D_s$, called *source domain*, to improve the quality of the results on the main domain $D_t$, called *target domain*.

Formally, in a classical supervised learning setting, given (i) the feature space *X* of training examples, (ii) the output space *Y*, and (iii) *n* training examples $\{(x_1,y_1),(x_2,y_2),\ldots ,(x_n,y_n)\}$, such that $x_i \in X$ and $y_i \in Y$, the goal is to learn a function *f*: $X \rightarrow Y$, that predicts the label/value of unseen, unlabeled examples.

Transfer learning differs from this formulation since it works on two different domains. Formally, given:

- the source and the target feature spaces $X_s$ and $X_t$;
- the output spaces $Y_s$ and $Y_t$;
- $n_s$ training examples $\{(x^s_1,y^s_1),(x^s_2,y^s_2),\ldots ,(x^s_{n_s},y^s_{n_s})\}$ s.t. $x^s_i \in X_s$ and $y^s_i \in Y_s$ for the source domain;
- $n_t$ training examples $\{(x^t_1,y^t_1),(x^t_2,y^t_2),\ldots ,(x^t_{n_t},y^t_{n_t})\}$ s.t. $x^t_i \in X_t$ and $y^t_i \in Y_t$ for the target domain;

the goal is to learn a function $f_t$: $X_t \rightarrow Y_t$ on the target domain, also exploiting the knowledge acquired by learning a function $f_s$: $X_s \rightarrow Y_s$ on the source domain.

**Figure 1:**
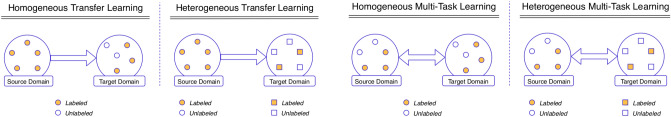
Homogeneous vs Heterogeneous transfer learning settings (left); Homogeneous vs Heterogeneous Multi-Task learning (right). The shape of the instances represents their feature space, while the arrow represents the direction of the transfer of knowledge between the domains.

In the literature, we can find several transfer learning approaches, which were designed either as general-purpose frameworks or as specific methods, tailored for solving specific tasks of an application domain. Such approaches can be classified according to two categories (see Fig. 1 (left)):

- *homogeneous*, when the source and the target domains are described according to the same feature space (i.e., $X_s = X_t$);
- *heterogeneous*, where there are no restrictions on the feature spaces (i.e., $X_s \ne X_t$).

The heterogeneous setting is clearly more difficult to handle, since it is necessary to design a strategy to transform both the feature spaces into a common feature space, or to make them comparable. For example, some heterogeneous transfer learning approaches^29–31^ assume that the source and the target domains are described with the same number of features and identify a shared feature subspace, where the difference between data distributions is minimized.

Another categorization of transfer learning methods^18^ distinguishes among:

- *instance-based* methods, that usually perform a reweighing of the source domain instances, that are then directly used during the training for the target domain (see^16,32,33^);
- *parameter-based* methods, that aim to transfer the knowledge through some parameters shared by the models learned for the source and the target domains (see^17,34,35^);
- *feature-based* methods, that perform knowledge transfer by identifying a shared feature space (see^29–31,36,37^).

Focusing on transfer learning approaches proposed in the field of bioinformatics, in the literature we can find a recent work that aims to classify breast cancer tumors, as Estrogen-Receptor-positive (ER-positive) or Estrogen-Receptor-negative (ER-negative), by exploiting two different data sources^38^. A different approach, based on deep learning, has been used for molecular cancer classification^39^, where the feature representation learned while classifying two tumor types also exploits information conveyed, in an unsupervised manner, by other tumor types. Breckels et al.^40^ propose to extend a state-of-the-art transfer learning framework to solve the predictive task of mouse protein sub-cellular localization.

To the best of our knowledge, all the cited methods require a fully labeled training set, or assume the presence of some negative examples, following the strategies (a) or (c) described in “Introduction” section. In our previous work^16,17^, we overcame this limitation by designing methods based on strategy (b), i.e., based on instance-weighting. In particular, these methods exploit the knowledge coming from the reconstruction of the mouse GRN for the reconstruction of the human GRN, in a homogeneous transfer learning setting. However, the main limitations of these methods are: (i) their inability to solve the gene network reconstruction task for both organisms simultaneously, and (ii) the homogeneous setting in which they work, that makes them hardly applicable if the gene relationships of the considered organisms are represented in different feature spaces.

The approach we propose in this paper exhibits the advantages of our previous work^16,17^, without their limitations. In particular, the proposed method works in a multi-task learning setting, which aims at solving both gene network reconstruction tasks simultaneously, and which can analyze the considered organisms in either homogeneous or heterogeneous feature spaces. Moreover, according to the second categorization^18^, our method falls in the category of *feature-based* transfer learning methods, since, as we will describe in “Methods” section, we identify a common feature space by exploiting the concept of genetic orthology.

### Multi-task learning

A specific sub-category of transfer learning methods is represented by *multi-task learning* methods, which aim at simultaneously solving the task for both the source domain $D_s$ and the target domain $D_t$. Such an advantage is not commonly present in standard transfer learning methods, which usually aim to facilitate or improve the task for the target domain only. On the contrary, multi-task learning approaches are able to optimize both tasks simultaneously, through multiple objective (or loss) functions, or their combination. The simultaneous consideration of the two tasks allows us to take into account possible bidirectional dependencies, which cannot be considered in single-task scenarios, even if a unidirectional transfer learning approach is applied multiple times.

Several complex machine learning applications have taken advantage of multi-task approaches, ranging from natural language processing^41^ and speech recognition^42^ to computer vision^43^ and GRN reconstruction^44^. To the best of our knowledge, there is only one multi-task learning method in the literature that is able to work in a positive-unlabeled setting^45^. However, it requires that some of the solved tasks are classical supervised tasks, where the training set also includes negative examples. This makes its application inappropriate in our case, since, in principle, both the gene network reconstruction tasks are posed in the positive-unlabelled setting. This is an important aspect, as well as a strong contribution provided by our method. Indeed, although we can find several multi-task methods that are able to work in the semi-supervised setting (e.g.,^46,47^), they cannot be easily adapted to work in the positive-unlabeled setting for both the considered gene network reconstruction tasks, due to the inherent additional challenges introduced by the absence of negative examples. Therefore, our method simultaneously exhibits all the following characteristics:

- it can work with no negative examples, using positive and unlabelled examples of both domains (*positive-unlabelled*);
- it is able to transfer the knowledge acquired in the reconstruction of a GRN of an organism for the reconstruction of the GRN of another organism (*transfer learning*);
- it can simultaneously reconstruct (see Fig. 1 (right)) the GRN of two organisms, i.e., the knowledge is transferred bidirectionally (*multi-task learning*).

It is noteworthy that multi-task approaches are closely related to multi-target prediction methods. Indeed, multi-target prediction refers to the (possibly simultaneous) prediction of multiple variables of interest for the same units of observation^48^. In our case, we are interested in predicting the existence of relationships between genes of two different organisms. Therefore, considering an output variable for each organism leads the considered task to be conceptually close to a multi-target prediction task (since it is in fact a multi-target link prediction task). This aspect will be clearer in the next section, where we describe how we employ our multi-target prediction approach to solve the network reconstruction task for two organisms simultaneously.

## Methods

In this section, we describe our method for simultaneous reconstruction of two GRNs in a multi-task learning setting. In particular, we will focus on the reconstruction of the human and mouse GRNs. To this end, we exploit the Predictive Clustering framework, that has proved its effectiveness in the presence of different forms of autocorrelation^49^, i.e., when objects that are close to each other appear more related than distant ones. This is useful in the context of network data, like the GRNs under study, where genes close in the network are expected to show a similar behavior or to participate in the same biological processes.

In particular, we exploit the Predictive Clustering Tree (PCT) method implemented in the system CLUS^50^. CLUS is a decision tree and rule induction system that unifies unsupervised clustering and predictive modeling. It has been employed in several recent works to solve multi-target prediction tasks for a single domain. The approach proposed in this paper can be considered the first attempt to employ the PCT multi-target prediction method implemented in CLUS to work in a multi-task learning setting, where the variables to be predicted are associated with two different tasks.

Multi-target prediction methods are generally categorized according to whether they build multiple *local* models, i.e., one model for each target variable, separately, or one *global* model, i.e., a single predictive model that is able to predict the whole set of target variables simultaneously. Global models are generally more effective than their local counterparts^51^, due to their ability to capture dependencies in both the input and the output spaces. In this paper we exploit the capability of CLUS to learn a global model, and we consider the degrees of existence of each gene interaction in the two organisms as two target variables of a multi-target regression task. This is achieved by representing the same examples of gene interactions, in the two organisms, in a common feature space. In particular, in order to find a match between the genes in the two organisms, we exploit the concept of orthologous genes, that are genes in different species that originated by vertical descent from a single gene of the last common ancestor.

Before describing in detail the proposed multi-task approach, we introduce some useful notions and formally define the problem that we solve. Let:

- $G_h$ (resp., $G_m$) be the set of considered genes for the human (resp., mouse) organism;
- $B_h \subseteq G_h \times G_h$ (resp., $B_m \subseteq G_m \times G_m$) be the set of (biologically validated) positive examples of gene relationships for the human (resp., mouse) organism;
- $orth_{hm}$: $G_h\rightarrow G_m$ (resp., $orth_{mh}$: $G_m\rightarrow G_h$) be a function that takes a human gene $g_h \in G_h$ (resp., a mouse gene $g_m \in G_m$) and returns the corresponding orthologous mouse gene (resp., human gene);
- $vec_h$: $G_h\rightarrow {\mathbb {R}}^{r}$ (resp., $vec_m$: $G_m\rightarrow {\mathbb {R}}^{q}$) be a function that returns the *r*-dimensional (resp. *q*-dimensional) vector of expression levels of a human (resp., mouse) gene;
- $^\frown$: ${\mathbb {R}}^{n_1}\times {\mathbb {R}}^{n_2}\rightarrow {\mathbb {R}} ^{n_1+n_2}$ be a function that takes as input two vectors in ${\mathbb {R}}^{n_1}$ and ${\mathbb {R}}^{n_2}$, respectively, and returns their concatenation in ${\mathbb {R}}^{n_1+n_2}$.
- $\tilde{vec_h}$: $G_h \times G_h \rightarrow {\mathbb {R}}^{2r}$ (resp., $\tilde{vec_m}$: $G_m \times G_m \rightarrow {\mathbb {R}}^{2q}$), be a function that takes as input two human (resp. mouse) genes and returns the concatenation of their vectors of expression levels, representing the features of their interaction. Formally, $\tilde{vec_h}(g'_h, g''_h) = vec_h(g'_h) ^\frown vec_h(g''_h)$ and $\tilde{vec_m}(g'_m, g''_m) = vec_m(g'_m) ^\frown vec_m(g''_m)$.

The task solved by our multi-task learning approach is to find two regression functions, namely:

- $f_h$: ${\mathbb {R}} ^{2r} \rightarrow [0, 1]$, that, given a pair of human genes $g_h' \in G_h$ and $g_h'' \in G_h$ represented through the feature vector of their interaction $\tilde{vec_h}(g_h', g_h'')$, returns a score representing the degree of certainty of the existence of the interaction between $g_h'$ and $g_h''$ in the human GRN.
- $f_m$: ${\mathbb {R}} ^{2q} \rightarrow [0, 1]$, that, given a pair of mouse genes $g_m' \in G_m$ and $g_m'' \in G_m$ represented through the feature vector of their interaction $\tilde{vec_m}(g_m', g_m'')$, returns a score representing the degree of certainty of the existence of the interaction between $g_m'$ and $g_m''$ in the mouse GRN.

Our goal is to learn both predictive functions simultaneously, by considering the degree of certainty of a given gene pair for the human and the mouse organisms as two different target variables of the same training example. It is noteworthy that this choice allows our method to capture possible dependencies that may exist in the output space (i.e., between the target variables). Specifically, we learn a single regression function $f_{hm}$ that takes as input a pair of genes represented according to the features related to both organisms, and returns the degree of certainty for both organisms. Formally:

1 $$\begin{aligned} f_{hm}: {\mathbb {R}} ^{2r+2q} \rightarrow [0, 1] \times [0, 1] \end{aligned}$$

Note that the construction of *all-in-one* training examples that can be used to learn a multi-target prediction model needs an additional step, i.e., the identification of a match between human genes and mouse genes. In the following subsections, we describe (i) the details of such a step, (ii) the strategy we adopt to solve the issues of the positive-unlabeled setting, (iii) the construction of the dataset used for learning the multi-target regression function $f_{hm}$, and (iv) the proposed predictive approach.

### Orthologous matching and construction of positive training examples

The first step of our method consists of the identification of possible matches between human and mouse genes. This step is necessary in order to represent each gene pair as a single training example, according to the features (i.e., expression levels) measured for both organisms.

**Figure 2:**
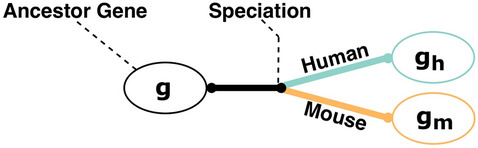
Speciation of an ancestor gene *g* into two genes $g_h$ and $g_m$. $g_h$ and $g_m$ are orthologs.

To this aim, we exploit the concept of gene orthology. Ortholog genes are genes of different species that are the result of the speciation of the same originating gene (see Fig. 2). Methodologically, we iterate over the human genes $g_m \in G_m$ and identify the corresponding orthologous gene in the mouse organism (Algorithm 1, Lines 2–27).

**Figure 3:**
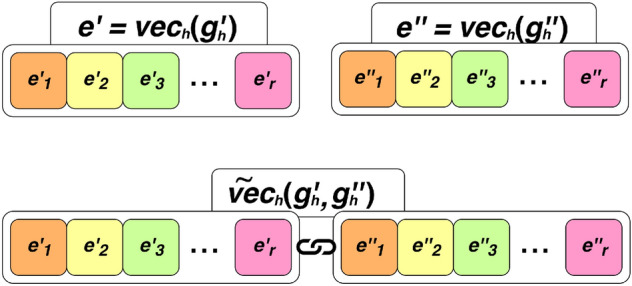
Concatenation of the expression vectors $e' = vec_h(g'_h)$ and $e'' = vec_h(g''_h)$ of the human genes $g'_h$ and $g''_h$ (the same holds for mouse genes).

At the end of this step, we obtain two new sets of genes: $G_{ho} \subseteq G_h$ consisting of the human genes that have orthologs in the set of mouse genes $G_m$, and $G_{mo} \subseteq G_m$ consisting of mouse genes that have orthologs in the set of human genes $G_h$.

The subsequent steps of the method work on the orthologous subsets of genes $G_{ho}$ and $G_{mo}$.

From a machine learning viewpoint, the set of genes corresponds to the set of nodes of the GRNs. However, our unit of analysis is a pair of genes, for which we want to estimate/predict the degree of existence of the interaction. Given that a human gene $g_h$ is described as a vector of expression levels $vec_h(g_h)$, we represent a pair of genes $g_h'$ and $g_h''$ as the concatenation of their feature vectors, i.e., $\tilde{vec_h}(g'_h, g''_h) = vec_h(g'_h) ^\frown vec_h(g'_h)$ (the same holds for the mouse organism). In this step, we build two sets of positive examples $P_h$ and $P_m$ (for human and mouse, respectively) by considering all the pairs of genes appearing in the set of validated interactions $B_h$ and $B_m$ (for human and mouse, respectively), for which we found a matching ortholog in the previous step. We then associate them with the corresponding feature vector (see Fig. 3 and Algorithm 1, Lines 8–12).

### Labeling of unlabeled examples

We recall that we work in the positive-unlabeled setting: together with the (positive) examples identified in the previous step, we also build a set of examples for which we do not have information about the existence of the interaction (unlabeled examples). Following the literature^17^, we assign a degree of certainty equal to 1.0 to positive labelled examples, and we estimate the degree of certainty for unlabeled examples according to their similarity with positive examples. Note that, differently from^17^, in this work we do not exploit such a similarity to assign a weight to the examples, but to assign a value to their target attributes.

Methodologically, we identify two sets of clusters $C_h$ and $C_m$, from the human and mouse positive interactions $P_h$ and $P_m$, respectively, that possibly represent different sub-concepts of existing gene interactions, and exploit them to estimate the value of the target variables of unlabeled examples.

In particular, given two feature vectors $u_h$ (for the human organism) and $u_m$ (for the mouse organism) for the same pair of genes, we compute the value of the target variables $t_h$ and $t_m$ as follows:

2 $$ \begin{aligned}& {\,} t_h(u_h) = \max _{c \in C_h} sim(u_h,cent(c)) \\&t_m(u_m) = \max _{c \in C_m} sim(u_m,cent(c)) \end{aligned}$$

where *cent*(*c*) is the feature vector of the centroid of the cluster *c*, and *sim*: ${\mathbb {R}}^{n} \times {\mathbb {R}}^{n} \rightarrow [0,1]$ is a vector similarity function. In this paper we use $sim(a,b) = 1 - \frac{\sqrt{\sum ^{n}_{i=1}\left( a_i - b_i \right) ^{2}}}{n}$, based on the Euclidean distance, after applying a min-max normalization (in the range [0, 1]) to all the features.

The identification of the clusters can actually be performed through any centroid-based clustering approach. In this paper we rely on the well-known *k-means* algorithm. Moreover, in order to optimally identify the number of clusters $k_h$ for the human organism and $k_m$ for the mouse organism, we use the silhouette cluster analysis^52^. Formally, we define a function *sil*: $P \rightarrow [1, 2, \ldots |P|]$, that, given a set of positive examples $P \in \{P_h,P_m\}$ and the clustering algorithm (in our case, *k-means*) returns the optimal number of clusters, according to the silhouette analysis. In Algorithm 1, this step is performed at Lines 13–14, whereas the exploitation of the identified clusters for computing $t_h$ and $t_m$ is performed at Lines 17–23.

After this step, the main issues of the positive-unlabeled setting are solved, since all the examples are associated to a (known or estimated) value for the target variables $t_h$ and $t_m$.

### Learning the predictive model

The final stage consists of learning the predictive model, in the form of a multi-target regression function, where the two target variables $t_h$ and $t_m$ represent the degrees of certainty of the existence of the interaction in the human and in the mouse organisms, respectively. With this aim, we build the final training set by concatenating, for each pair of genes for which we identified an ortholog match, (i) the 2*r*-dimensional feature vector associated to the human organism, (ii) the 2*q*-dimensional feature vector associated to the mouse organism, (iii) the target variable $t_h$, and (iv) the target variable $t_m$, leading to training examples represented in ${\mathbb {R}}^{2r+2q}$, associated to two target variables (see Algorithm 1, Line 24).
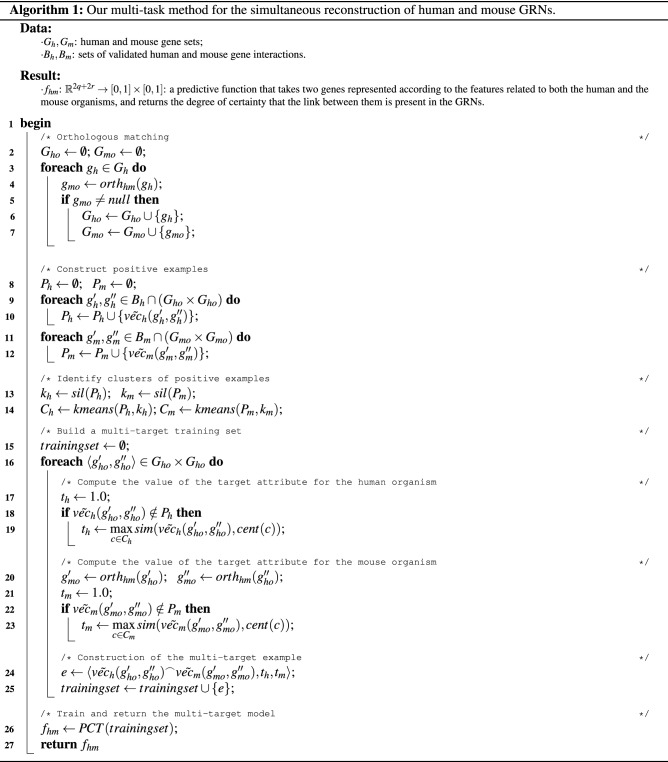


We learn the predictive model with CLUS^50^, that is based on Predictive Clustering Trees (PCTs). We induce PCTs through a standard approach for the top-down induction of decision/regression trees, that takes as input a set of training examples and returns the induced tree. The heuristics adopted to select the best tests of the internal nodes of the tree is the reduction of variance achieved by partitioning the examples according to such a test. The maximization of the variance reduction leads to maximizing the cluster homogeneity and, therefore, to improving the predictive performance. Therefore, the considered heuristic is formally defined as $$ Var_E(t_h,t_m)=Var_E(t_h) + Var_E(t_m), $$, where $$Var_E(t_h)$$ (resp., $$Var_E(t_m)$$) is the variance observed on the target attribute $$t_h$$ (resp., $$t_m$$) on the set of examples *E* falling in a given node of the tree.

This means that the variance reduction, used to identify the best candidate split in the tree construction, is computed as:

3 $$\begin{aligned} h = Var_E(t_h,t_m) - \left( Var_{E'}(t_h,t_m) + Var_{E''}(t_h,t_m)\right) \end{aligned}$$

where $E, E', E''$ are the sets of examples in the parent, left and right child nodes, respectively.

The final learned model will represent the function $f_{hm}$: ${\mathbb {R}}^{2r+2q} \rightarrow [0, 1] \times [0, 1]$ that can be employed to predict the degree of certainty for all the unlabeled examples for both considered organisms. Therefore, as explained in “Methods” section, $f_{hm}$ acts like the two functions $f_h$ and $f_m$ simultaneously, with the advantage of being able to capture dependencies in the output space.

## Experiments

In this section, we present the results of our experimental evaluation. All the experiments were performed on a server equipped with a 6-cores CPU @ 3.50Ghz and 128GB RAM. In the following subsections, we first describe the considered competitor systems, the datasets and the experimental setting. Finally, we present and discuss the obtained results.

### Competitor approaches

We compared our method with the following competitor approaches:

- **TJM**^29^, that reduces the difference between the two domains by identifying a match between their features and by reweighting the instances to construct a new reduced/shared feature representation;
- **BDA**^31^, that adaptively leverages the importance of the marginal and conditional distribution discrepancies between the two domains;
- **JGSA**^30^, that learns two coupled projections, that are exploited to project the source and the target domain data into low-dimensional subspaces, where the geometrical and the distribution discrepancies are minimized;
- **no_transfer**, that is the single-domain variant of our approach, which reconstructs each single GRN independently.

TJM, BDA, and JGSA are feature-based transfer learning methods that are able to share the knowledge between (also) heterogeneous source and target domains, as long as they are described with the same number of features. On the other hand, the no_transfer approach can be considered a baseline, that allows us to evaluate the positive contribution of the multi-target approach proposed in this paper or, conversely, to evaluate the possible presence of *negative transfer* phenomena^53,54^, where the use of knowledge coming from other domains actually compromises the quality of the reconstruction.

### Datasets

We built the dataset by downloading a compendium of microarray data of both human (Platform ID: GPL570) and mouse (Platform ID: GPL1261) organisms from Gene Expression Omnibus—GEO (www.ncbi.nlm.nih.gov/geo/), a publicly available web repository hosted by the National Center for Biotechnology Information (NCBI). In total, 174 and 161 raw CEL files related to 54,675 and 45,101 control probesets of 6 different organs were downloaded for human and mouse organisms, respectively (see Supplementary Table S1 for a complete list of accession numbers). More specifically, the 174 CEL files for the human organism are distributed as follows: 17 for bone marrow, 37 for brain, 4 for heart, 7 for liver, 45 for lung, and 64 for skin. On the other hand, the 161 CEL files of the mouse organism are distributed as follows: 14 for bone marrow, 8 for brain, 8 for heart, 124 for liver, 4 for lung, and 3 for skin.

**Figure 4:**
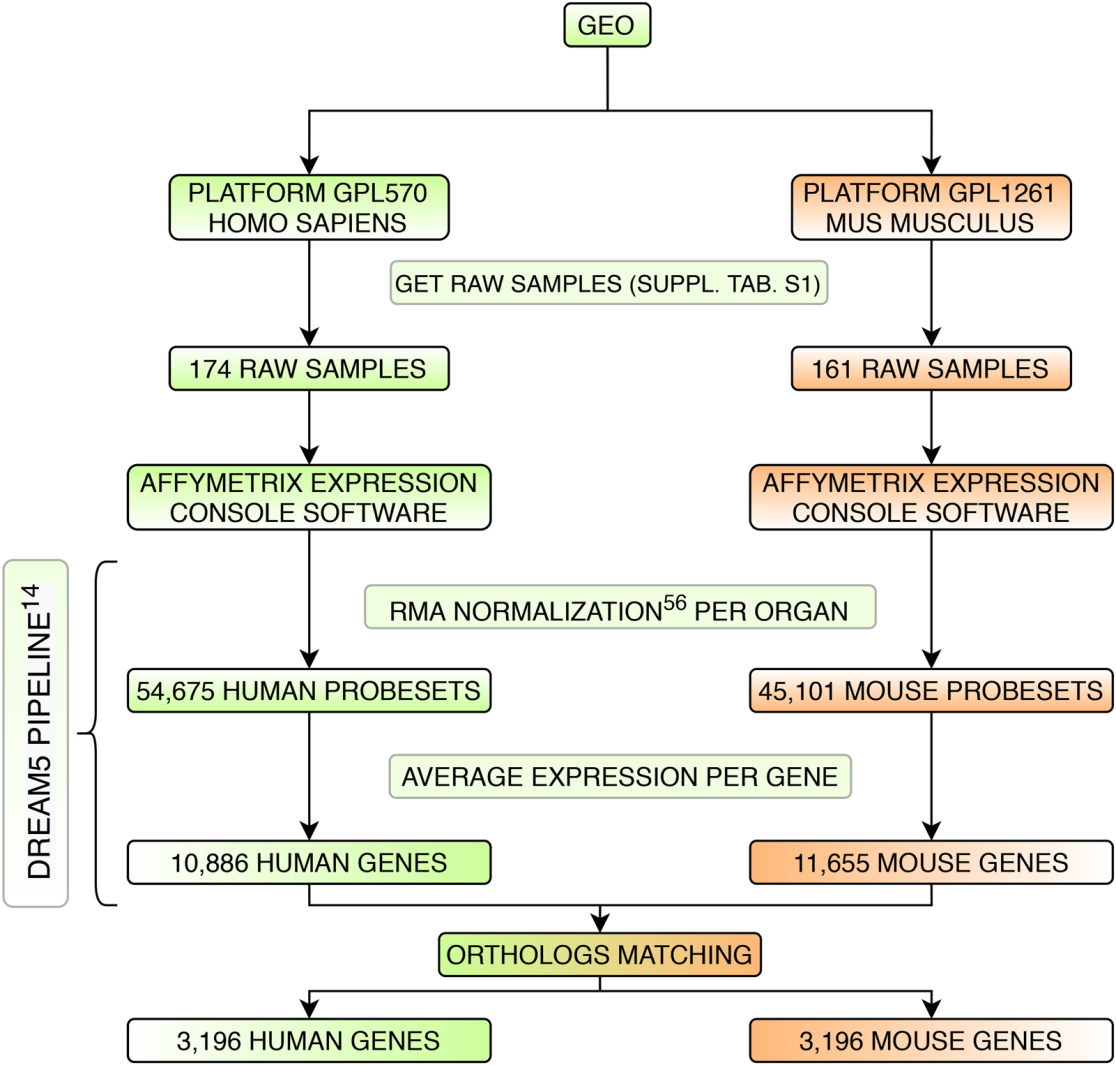
Graphical overview of the pipeline followed to identify the genesets and their gene expression levels.

We processed the data following the workflow proposed in the DREAM5 challenge^14^ (see Fig. 4 for a graphical overview of the followed pipeline). In particular, we performed the Robust Multi-array Average (RMA)^55^ normalization through Affymetrix Expression Console Software as one batch per organ. Data were background adjusted, quantile normalized, median polished and log-transformed. The mapping from Affymetrix probeset IDs to gene IDs led to a total of 10,886 human genes and to 11,655 mouse genes.

After the ortholog matching (see “Orthologous Matching and construction of positive training examples” section), we obtained a reduced set of 3, 196 genes for both organisms. The strategy adopted to perform the ortholog matching was based on the gene symbol, that corresponds between homo sapiens and mus musculus organisms, except for differences in the capitalization^56^. Alternative solutions may have been adopted, mostly based on explicit lists of ortholog genes (see, for example, the OMA orthology database^57^), but the strategy based on the gene symbol provided us 153 additional matches. Note that the set of 3,043 genes was included in the set of 3,196 genes we considered.

The dataset of possible relationships between genes was built by considering all the possible pairs of genes (more than 10 million, excluding the self-links), each associated with the concatenation of the feature vectors of the involved genes (following the strategy described in “Methods” section). This step led to 348-dimensional vectors for human gene pairs and to 322-dimensional vectors for mouse gene pairs. We exploited the database BioGRID^20^ as the source of known validated interactions (i.e., to define the sets $B_h$ and $B_m$), while the remaining possible relationships were considered unlabeled. In Table 1 we report a summary of the quantitative characteristics of the considered dataset.

**Table 1:** Quantitative characteristics of the dataset.

	Human	Mouse
Probeset	54,675	45,101
Geneset	10,886	11,655
Orthologous geneset	3,196	3,196
Gene features	174	161
Gene-pair features	348	322
Positive examples	3,970	3,970
Unlabeled examples	75,430	75,430

**Table 2:** Number of examples for each variant of the dataset.

Dataset	50%	40%	30%	20%	10%	5%
Positive	3,970	3,970	3,970	3,970	3,970	3,970
Unlabeled	3,970	5,558	9,263	15,880	35,730	75,430

**Figure 5:**
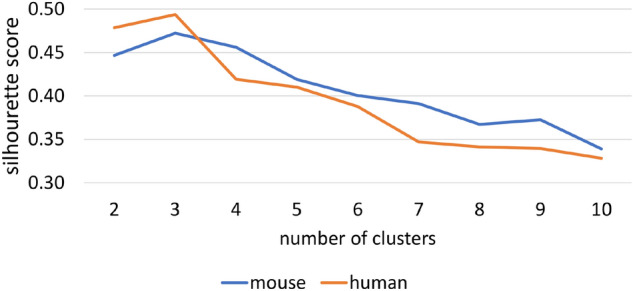
Silhouette score for the human ($k_h$) and mouse ($k_m$) organisms.

Since some competitor systems, even if they are able to work in the heterogeneous transfer learning setting, require the number of features of all the domains to correspond, we also built a homogeneous version of the dataset. In particular, we aggregated the features associated to each organ (by averaging their value), leading to a homogeneous dataset consisting of 6 features per gene, for both the human and the mouse organisms.

Finally, we evaluated the robustness of the proposed method with respect to the number of unlabeled examples used during the training phase. With this aim, we considered a sample of maximum 75, 430 unlabeled examples and we built 6 variants of the datasets, with a different ratio of the number of positive examples over the unlabeled examples (see Table 2). It is worth mentioning that the comparison with competitors has been performed on the smallest version of the dataset (i.e., with the ratio 50%), because they were not able to complete the experiments with larger datasets without incurring in RAM exhausting errors on our servers. Indeed, while our approach is based on a top-down induction of regression trees, that is generally efficient, competitor methods are mainly based on matrix computations and easily exhausted the RAM on our servers even with the considered reduced dataset.

### Experimental setting

The results have been produced according to a 10-fold cross-validation approach, where each fold consists of 9/10 of the positive examples for training and 1/10 of the positive examples for testing, while all the unlabeled examples are considered for both training and testing. The preliminary estimation of the optimal value of $k_h$ and $k_m$ for our method led to the results reported in Fig. 5. Accordingly, we considered the best configurations $k_h = 3$ and $k_m = 3$ for the subsequent experiments.

Since we work in the positive-unlabeled learning setting where no negative examples are available^16,17,58^, we evaluate the performance of different methods in terms of recall@*k* and the area under the recall@*k* curve (AUR@*K*). The recall@k measure is defined as $\frac{TP_k}{TP+FN}$, where $TP_k$ is the number of returned true positive interactions, within the first top-*k* interactions, and $(TP+FN)$ corresponds to the number of positive examples in the testing fold. This formula allows us to evaluate the ability of the method to put reliable interactions on the top part of the returned ranking. The recall@*k* curve is a curve representing the recall@*k* with varying values of *k*, while the AUR@*K* measure corresponds to the area under such a curve.

We also report the results in terms of the area under the ROC curve (AUROC) and the area under the precision-recall curve (AUPR). Note that the recall@*k* and the AUR@K measures do not introduce any (possibly wrong) bias in the ground truth, while in the computation of the AUROC and AUPR measures, it is necessary to assume the unlabeled examples as negative examples.

### Results

**Figure 6:**
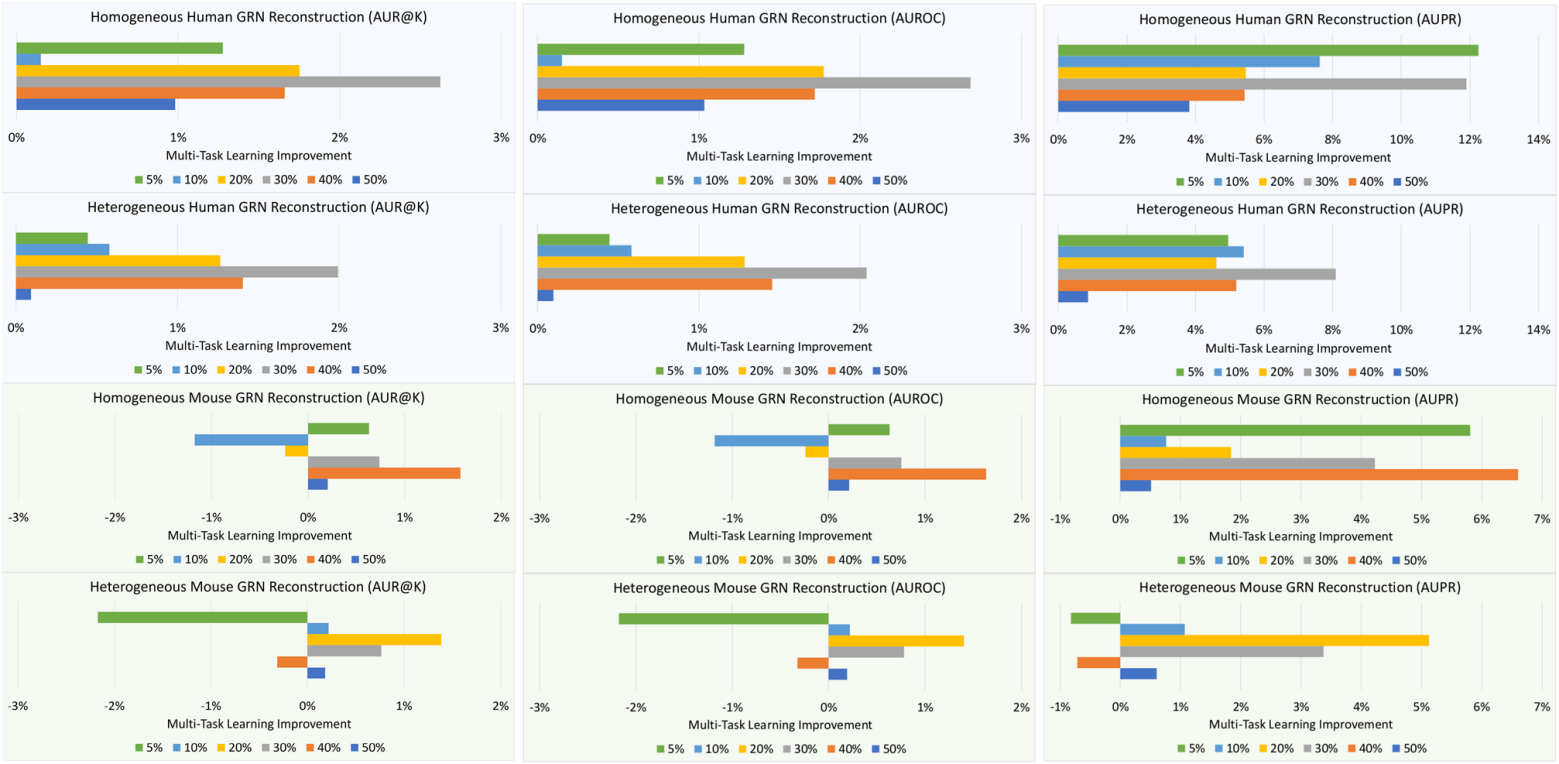
Improvement achieved by our approach with respect to no_transfer, in terms of AUR@K, AUROC and AUPR.

Figure 6 depicts the improvement obtained by our approach with respect to the baseline no_transfer, in terms of the AUR@K, AUROC and AUPR measures, for both the homogeneous and heterogeneous datasets, and with respect to both organisms.

The charts show that the proposed approach provides a marked improvement over the single-domain counterpart in the reconstruction of the human GRN. Such an advantage is evident for all the variants of the dataset, i.e., for all the considered labeled/unlabeled ratios. On the other hand, the reconstruction of the mouse GRN appears to exploit the knowledge about the human GRN only with higher labeled/unlabeled ratios. This may suggest that the mouse organism can be considered an appropriate model organism for the study of the human GRN, but the contrary may hold to a lesser degree, i.e., only when there is a sufficient amount of biologically validated information.

**Table 3:** Summary of settings for which the multi-task approach provided an improvement over the baseline.

Measure	50%	40%	30%	20%	10%	5%
**Homogeneous human GNR**						
AUROC	V	V	V	V	V	V
AUPR	V	V	V	V	V	V
AUR@K	V	V	V	V	V	V
**Heterogeneous human GNR**						
AUROC	V	V	V	V	V	V
AUPR	V	V	V	V	V	V
AUR@K	V	V	V	V	V	V
**Homogeneous mouse GNR**						
AUROC	V	V	V	V	X	V
AUPR	V	V	V	V	V	V
AUR@K	V	V	V	X	X	V
**Heterogeneous mouse GNR**						
AUROC	V	X	V	V	V	X
AUPR	V	X	V	V	V	X
AUR@K	V	X	V	V	V	X

**Figure 7:**
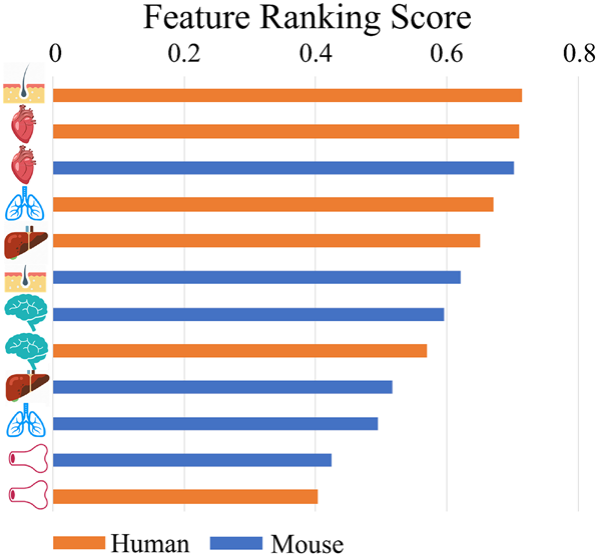
Ranking of the features in the homogeneous setting.

**Figure 8:**
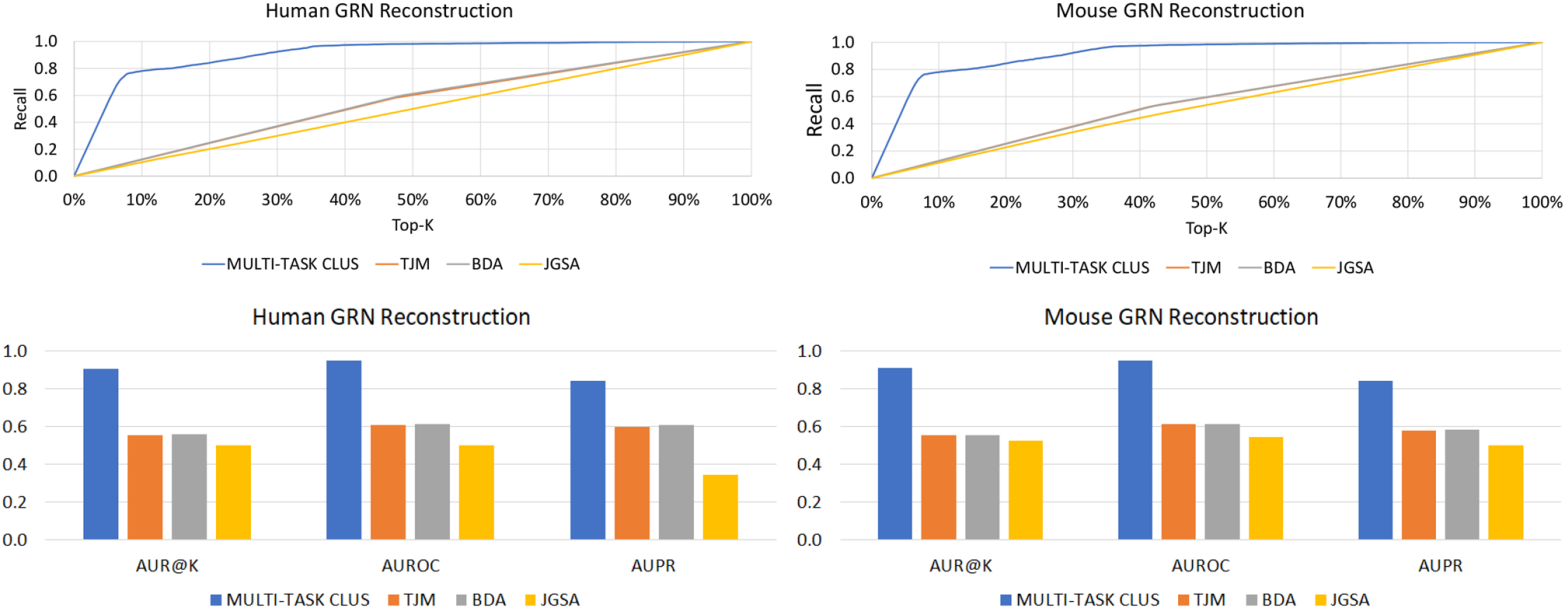
Recall@k (charts in the top) and AUR@K, AUROC and AUPR (charts in the bottom) results, obtained by our method (referred to as MULTI-TASK CLUS here) and its competitors.

In general, for both organisms, the higher the labeled/unlabeled ratio, the better the quality of the reconstruction. This is an expected result, since the unlabeled examples could belong to clusters of positive examples that were not properly represented/observed in the set of positive examples, due to their limited availability. Despite such an expected result, the reconstruction performed with our multi-task approach in most of the cases provides an advantage, even with very low labeled/unlabeled ratios. The few cases in which there is not an improvement occur in the reconstruction of the mouse regulatory network. This may indicate that it is easier and more natural to exploit the knowledge coming from a general/simple organism to describe a complex organism, rather than vice versa. In Table 3 we show a summary of the settings where our multi-task approach provided an improvement over the baseline.

Moreover, as explained in “Competitor approaches” section, we compared our results with those obtained by three state-of-the-art approaches. In Fig. 8 we present the results in terms of recall@k, AUR@K, AUROC and AUPR. Observing the figure, it is clear that our approach outperforms all the competitors by a large margin for both the considered organisms.

We also performed a qualitative evaluation of the networks reconstructed by our system. For this specific analysis, we considered the largest version of the dataset, containing 3, 970 positive examples and 75, 430 unlabelled examples (see the variant $5\%$ in Table 2). Since the experiments were performed using 10-fold cross validation, resulting in 10 different rankings (one for each fold), we averaged the scores and analyzed the resulting ranking. We then selected the top 10, 000 ranked interactions for both organisms, we computed some topological measures (see Supplementary Tables S2 and S3 for a detailed overview) and we identified the hub genes, by selecting the top-$10\%$ of genes with the highest numbers of regulated genes^59^. Among them, we selected the 352 genes appearing as hubs for both the considered organisms, and we plotted the subnetworks involving each of them, emphasizing the interactions that were present in BioGRID (in black) and those that were predicted by our system (in green). It is noteworthy that the subnetworks of the first 60 hub genes are identical, in both known and predicted interactions, between the two organisms. This confirms the ability of our system in catching cross-organism similarities and in predicting the existence of interactions that appear coherent among the organisms.

**Figure 9:**
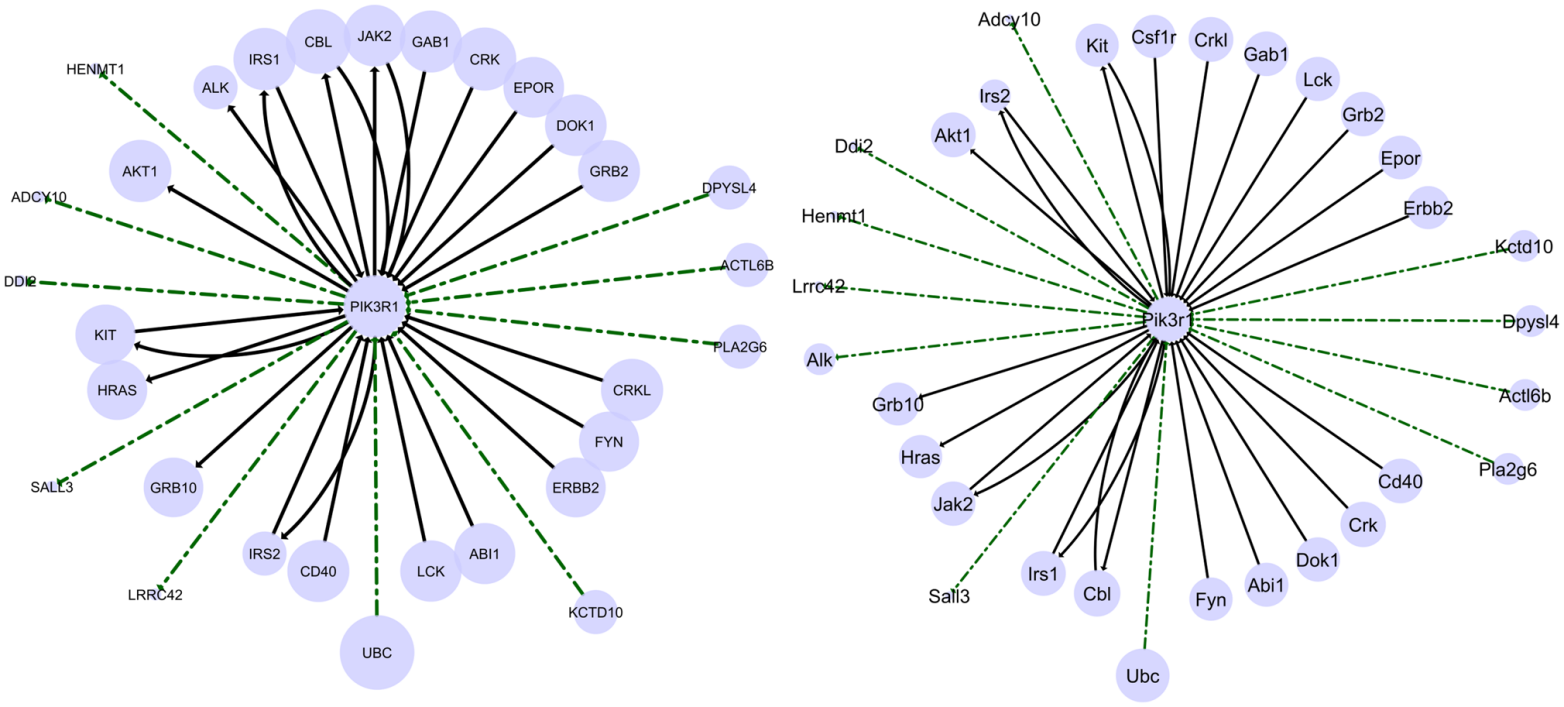
The subnetwork identified for the human hub gene PIK3R1 (on the left) and the mouse hub gene Pik3r1 (on the right). The size of the circle of a gene represents the number of genes regulated by such a gene. Known interactions (from BioGRID) are represented as black lines, while interactions predicted by our system are represented as green dotted lines.

In Fig. 9 we depict the first subnetwork that shows some differences between the organisms (i.e., the 61th in the ranking). This is the case of the human gene PIK3R1 (resp., Pik3r1 for the mouse organism). In this case, we can observe 5 (resp., 6) predicted regulated genes for the human (resp., for the mouse) organism and 5 (resp., 5) predicted genes regulating PIK3R1 (resp., Pik3r1). Specifically, it is noteworthy that the interaction Pik3r1 $\rightarrow$ Alk has been inferred by our method, but is not covered in BioGRID. On the other hand, the interaction Csf1r $\rightarrow$ Pik3r1 is present in BioGRID for the mouse organism, but our method did not suggest the corresponding interaction for the human organism, that is actually absent in BioGRID (preventing, therefore, a possible false positive). This confirms that, although our method exploits the knowledge coming from the simultaneous reconstruction of the regulatory networks of both the organisms, it does not merely mimic the behavior observed on an organism on the other one. On the contrary, it is able to catch possible differences and asymmetries.

Finally, we performed an additional analysis regarding the importance of the considered features. In particular, focusing on the homogeneous dataset, we aimed to identify the most relevant organs (i.e., those most relevant to our method during the learning of the multi-target regression model), following the approach of Petković et al.^60^. In Fig. 7 we show the obtained ranking, where we can observe that the features associated to the human skin and heart, together with those associated to the mouse heart and lung, have been detected as the most relevant ones for the gene network reconstruction task. In contrast, it seems that features related to bone marrow (for both organisms) did not provide any relevant contribution. In the middle, we find the features closely related to the brain (for both organisms), the human liver and the human lungs. This behaviour is probably motivated by the fact that some organs show more similar properties between the two organisms, or are better connected through orthologous genes, than others. It is noteworthy that these findings can be profitably exploited to focus future work, where larger sets of samples, related to the organs that provide a higher contribution, can be adopted.

## Conclusion

Several computational approaches, mainly based on machine learning methods, can be employed for the reconstruction of GRNs. However, existing gene network reconstruction methods suffer when the number of labeled examples is low, especially when no negative examples are available. In this paper we have proposed a method that overcomes these limitations. Our approach is able to simultaneously reconstruct the GRN of two organisms, by exploiting a multi-target regression approach that, in conjunction with the concept of gene orthology, is able to natively work in a positive-unlabeled learning setting.

The experiments show that our approach is able to really “transfer” knowledge extracted from an organism and profitably use it in another organism. Moreover, the proposed multi-target positive-unlabeled learning algorithm outperforms both its single-task counterpart and three state-of-the-art transfer learning approaches in the reconstruction of both GRNs.

As future work we plan to define a more general approach to map the examples in the considered domains, so that it may be adopted in multiple, even non-biological, applications. Moreover, while in the present paper we presented our novel approach and evaluated its effectiveness, compared with state-of-the-art methods, we plan to extend the experiments to larger datasets, also considering different pipelines.

## Supplementary information

Supplementary Information

Supplementary Information

Supplementary Information
